# Preservation of fecal glucocorticoid metabolites and immunoglobulin A through silica gel drying for field studies in horses

**DOI:** 10.1093/conphys/coz065

**Published:** 2019-10-27

**Authors:** Konstanze Krueger, Isabell Marr, Andrea Dobler, Rupert Palme

**Affiliations:** 1 Department Equine Economics, Nuertingen-Geislingen University, Faculty Agriculture, Economics and Management, Neckarsteige 6-10, 72622 Nürtingen, Germany; 2 University of Regensburg Zoology/Evolutionary Biology, Universitätsstraße 31, 93053 Regensburg, Germany; 3 Department of Behavioral Physiology of Livestock, University of Hohenheim, Garbenstr. 17, 70599 Stuttgart, Germany; 4 Department of Biomedical Sciences, University of Veterinary Medicine, Veterinär-Platz 1, 1210 Vienna, Austria

**Keywords:** Drying on silica gel, *Equus caballus*, fecal glucocorticoid metabolites, field studies, horse, immunoglobulin A, non-invasive sampling

## Abstract

Non-invasive methods enable stress evaluation through measuring fecal glucocorticoid metabolites (FGMs), and immunoglobulin A (IgA) in the feces avoiding stressful blood drawing or stressful restraining of animals in the field. However, FGMs and IgA are mostly analysed in freshly frozen samples, which is difficult when fresh samples cannot be frozen immediately or frozen samples cannot be stored or transported. Good results were also derived from air-dried fecal samples, which are hampered by unstable air humidity in the field. These difficulties may be overcome, when drying of samples could be induced with colorless silica gel (SiO_2_) granules in a secure set-up, such as an air tight tube. We determined the speed of drying 1.5 g of a fresh fecal sample from six horses on air and on silica gel. Furthermore, FGMs and IgA were analysed in differently stored subsamples from 12 horses: in frozen fecal samples, in air- or silica gel-dried samples stored for 1 day and for 7 days, and in wet fecal samples kept in a tube at room temperature for 7 days. FGM levels remained stable in feces dried on air or on silica gel for 7 days, whereas IgA quantities showed a significant loss. Under field conditions, when freezing or transporting the frozen samples is not possible and humidity hampers air drying, drying samples on silica gel in air tight tubes appears to be very helpful and reliable for analysing FGMs.

## Teaser

Stability of stress hormone metabolites, fecal glucocorticoid metabolites (FGMs), and immunoglobulin A (IgA) was evaluated in horse feces dried in an air tight tube on silica gel, for improving stress analysis with fecal samples collected under field conditions with uncontrollable humidity. IgA showed a significant loss, but FGMs were stable and appeared to be reliably preserved.

## Introduction

The present study investigates whether two stress parameters, fecal glucocorticoid metabolites (FGMs) and immunoglobulin A (IgA), are well preserved in dried samples taken from fecal heaps in a herbivore, such as the horse (McGhee and Mestecky, 1990; Möstl and Palme, 2002; Palme, 2019).

Secretion of glucocorticoids (adrenal stress hormones) is enhanced when stressful events activate the hypothalamic–pituitary–adrenal axis. Glucocorticoids stimulate the carbohydrate, protein, and lipid metabolism, as well as the immune response (Möstl and Palme, 2002). As prolonged stress may cause glucocorticoid levels to decrease to baseline values (Thun and Schwarz-Porsche, 1994; Pawluski *et al.,* 2017), a combination with other parameters (such as immunological ones) is helpful. The immune system responds to stress with an increased production of immune cells in acute stress situations. Under chronic stress, the immune system will be depressed and the production of immune cells will be significantly inhibited and decline below baseline values (Siegel, 1987; Herbert and Cohen, 1993). IgA appears to be a suitable parameter for measuring the immune response in the gut, as it constitutes the main antibody in local immune defence in many mammals. IgA inhibits the binding of bacteria and viruses at the outer epithelial layers and reduces infections (McGhee and Mestecky, 1990). FGM and IgA quantities may increase with age as reported for dogs (IgA: Zaine *et al.*, 2011) and may differ between sexes (FGMs: Gorgasser *et al*., 2007; IgA: Weber-Mzell *et al*., 2004) and between individuals (FGMs: Möstl *et al*., 1999; IgA: Paramastri *et al*., 2007).

Glucocorticoid metabolites (GMs) and IgA can be analysed in the blood or via non-invasive sampling in horse feces (FGMs: Flauger *et al*., 2010; review: Palme, 2012, 2019; IgA: May, 2007). For several species, non-invasive sampling provides reliable measurement of FGMs and IgA avoiding stressful blood drawing and enables field researches to collect samples without restraining the animals (Sheriff *et al*., 2011). In the past, FGM and IgA quantities were usually analysed in frozen fecal samples (IgA and FGM: rats: Eriksson *et al*., 2004; horses: May, 2007; mice: Moon *et al*., 2015), in fresh fecal samples (only IgA: dogs: Zaine *et al.*, 2011), or in fecal samples stored on alcohol (only FGM: Palme *et al.*, 2013). Freezing prevents further bacterial metabolism of glucocorticoid metabolites (Möstl and Palme, 2002) and the destabilization of IgA (Hau *et al*., 2001) best. However, immediate freezing may not be possible in the field or the transportation of frozen samples may be too difficult and costly.

Some studies acquired good results for conserving FGMs and IgA by drying samples in the lab (FGMs: review: Palme *et al*., 2013; cats: Ramos *et al*., 2013; macaques: Gholib *et al*., 2018; IgA: human: Vetvik *et al*., 1998). In humans, IgA quantities are reduced through air drying but can reliably be quantified by extrapolating IgA amounts from dried to frozen or fresh samples (Vetvik *et al*., 1998). We tested whether the preservation of FGMs and IgA through drying could be achieved in the field. We wondered whether drying of fecal samples can be performed with colorless silica gel (SiO_2_) granules in a water tight, secure set-up, such as an air tight tube. Adding silica gel for preservation through water absorption is widely applied in scientific fields, such as botany (Chase and Hills, 1991) and for genetic analysis (Taberlet *et al*., 1999; Murphy *et al*., 2002; Engelhardt *et al*., 2017). Whether fecal FGM and IgA quantities are stable in samples dried on silica gel over time has not been evaluated.

The horse is a good model organism for testing the stability of FGMs and IgA in feces dried on silica gel as methods for their measurement have been well established (FGMs: Möstl *et al*., 1999; Merl *et al*., 2000; Flauger *et al*., 2010; Palme, 2012; Wolter *et al*., 2014, Yarnell *et al*., 2015) or at least reported (IgA: Vaerman *et al*., 1971, May, 2007). In the present study, we collected fresh fecal samples from 18 warmblood horses. We measured the speed at which given fecal quantities can be dried under lab conditions on air and in an air tight tube on silica gel. We compared concentrations of FGMs and IgA in frozen samples with those of air or silica gel-dried samples after 1 and 7 days and in samples kept in tubes at room temperature for 7 days without drying. We aimed to evaluate whether IgA and FGMs can be preserved reliably when dried on silica gel. We assumed (i) that IgA will destabilize significantly, but reproducibly when dried with silica gel, and (ii) that FGM quantities dried on silica gel should be well preserved and stable when the drying is as effective as air drying under controlled conditions in the lab.

## Materials and Methods

### Animals and Location

Eighteen horses (9 mares, 8 geldings, and 1 stallion) were used for the study. All 18 were warmblood horses and were aged between 1 and 27 years (median = 8 years). The feces for the study were sampled at the stable of the University of Applied Sciences Nürtingen-Geislingen and an adjacent private stable. Horses were given hay *ad libitum* and amounts of oat feed and mineral supplements were adjusted to individual needs.

### Sample collection and preservation

Single samples were collected from six horses in April, June, and July 2016 and from further 12 horses in November 2017 between 7:00 a.m. and 8:00 a.m. from dung heaps defecated within 1 hour before collection. Pool samples from five different locations of the dung heap were collected with one-way gloves, stored in unused freeze bags, and homogenized by kneading for 2–3 minutes.

### Speed of air drying and drying on silica gel

**Figure 1:**
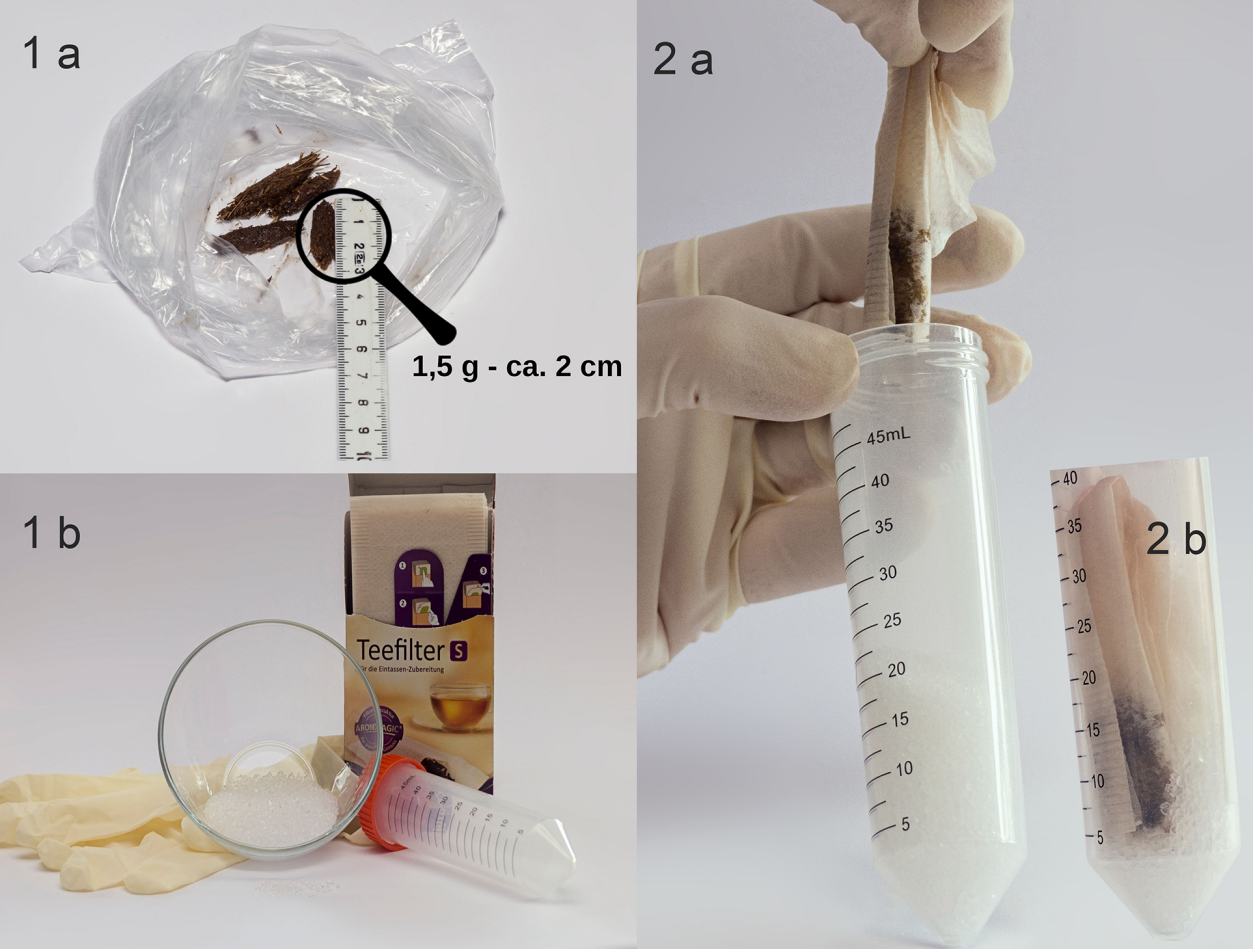
Drying fecal samples on silica gel. 1a, From the fecal pool samples, an aliquot of 1.5 g was taken and formed to an approximate 2-cm-long roll to increase surface for faster drying. 1b, One way gloves, 20 mL of colorless silica gel granules, a small paper tea bag, and a 50-mL tube were used. 2a, The samples were placed in a tea bag, and the tea bag coiled, its ends folded, and placed in a tube with 20 mL of silica gel. 2b, The lid was screwed on the tube with the sample and tilted a couple times until silica gel was all around the tea bag with the sample.

From the samples of the six horses collected in 2016, we evaluated how fast 1.5 g of feces lost humidity under the following conditions: (i) when spread out in a petri dish and air dried at room temperature (20°C) in a lab without air conditioner or controlled air flux (air drying = AD) and (ii) when given in a paper tea bag and dried in an air tight tube on 20 mL of colorless silica gel granules (silica gel drying = SD; Fig. 1). For each horse, we measured the weight loss (i.e. humidity loss) of the samples after 12, 24, 48, and 72 hours of drying.

### Preservation method

The samples taken from 12 horses in 2017 were aliquoted for all preservation procedures and the following preservation methods were applied: (i) 1.5 g of fresh feces were frozen at −20°C (frozen sample = FR), (ii) 1.5 g of fresh feces were placed in an airtight tube and kept for 7 days at room temperature without drying (wet sample room temperature = WR), (iii) 1.5 g of fresh feces were spread out in a glass petri dish and air dried for 7 days at room temperature (air dry = AD), or (iv) 1.5 g of fresh feces were placed in a paper tea bag and dried for 7 days in an air tight tube on 20 mL of colorless silica gel granules (silica gel drying = SD; Fig. 1).

## Analyses of fecal samples

### Fecal glucocorticoid metabolites

Glucocorticoid metabolites were extracted from horse feces with the simplified method described by Flauger *et al*., (2010). We used 0.5 g of wet feces. In the dried samples, we balanced the weight loss from drying by weighting the sample after drying and using the corresponding dry mass to 1.5 g of fresh feces for each sample (for complete data, see Supplementary Table S2; for calculation for balancing weight loss from drying, see Supplementary File S1).

Thereafter, 0.5 g of wet feces (or the balanced dried fecal samples) plus 1 mL of water and 4 mL of methanol were vortexed for 2 minutes, kept at room temperature for 15 minutes, and vortexed again for 1 minute. The methanolic suspension was centrifuged. An aliquot of the supernatant was diluted in assay buffer and frozen until analysis. FGMs were quantified using an 11-oxoetiocholanolone enzyme immunoassay (EIA; for details, see Möstl and Palme, 2002), which has been validated for horses (Flauger *et al*., 2010). All differently stored subsamples of a horse were analysed (in duplicate; coefficient of variance (CV): <10%) consecutively on two microtitre plates in total (6 horses per plate).

**Figure 2:**
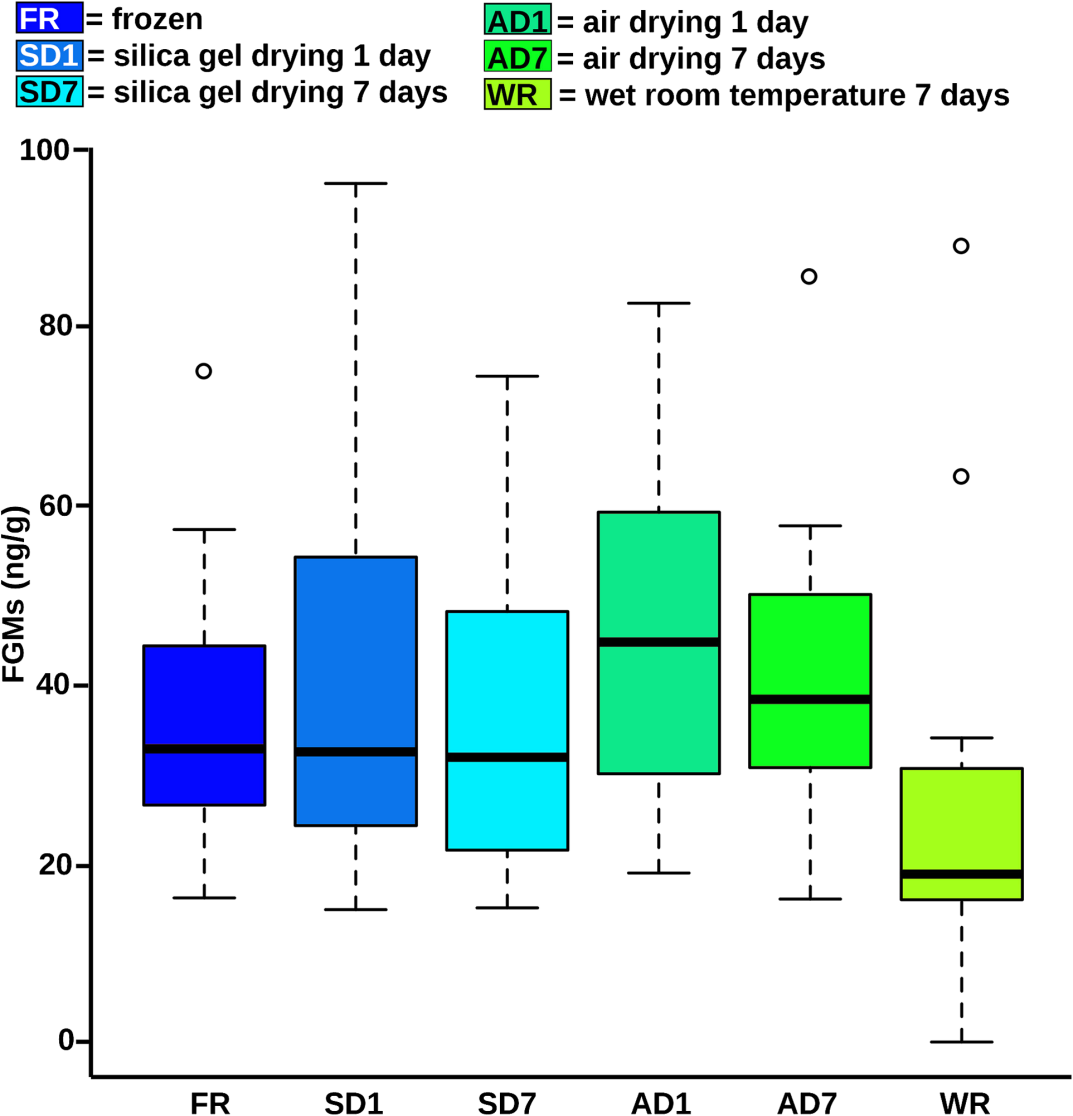
Concentrations of FGMs. The whisker boxplots depict the data of the particular preservation method. The line in the box represents the median and the circles above the box represent outliers. For full statistical data, see Supplementary File S4.

**Figure 3:**
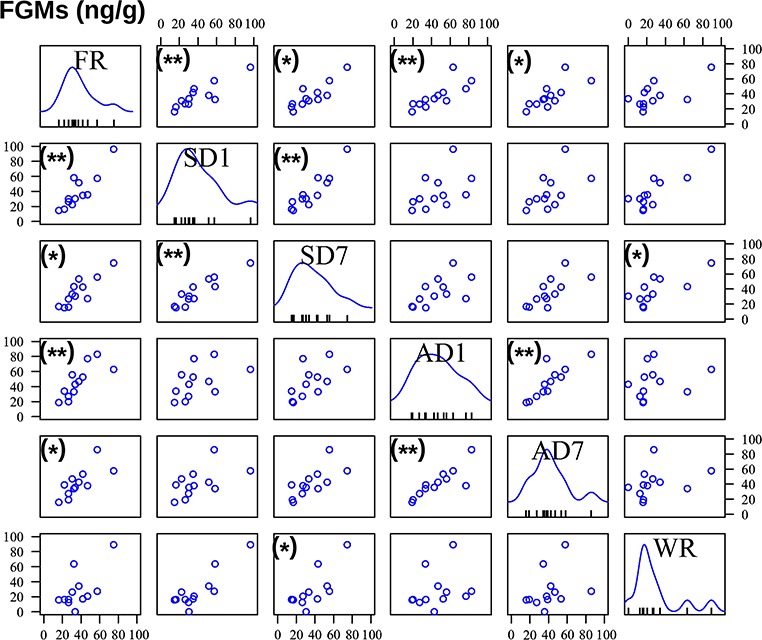
Correlations of FGM concentrations between preservation procedures. On the diagonal from the upper left to the lower right, the frequency distributions of FGM concentrations are shown for the 12 samples of each preservation procedure: FR = frozen samples, SD1 = silica gel-dried sample 1 day, SD7 = silica gel-dried samples 7 days, AD1 = air-dried sample 1 day, AD7 = air-dried samples 7 days, WR = wet sample at room temperature 7 days. FGM concentrations are given in ng/g for the *x* axis at each column. The 12 lines on the *x* axis within each preservation graph indicate the FGM concentration of each of the 12 samples. The scatterplots depict the correlations between FGM concentrations of pairs of preservation procedures (for full statistical data, see Supplementary File S4). For the correlations, *y* and *x* axes quantify the concentrations of FGMs in the respective column (*x* axis) or row (*y* axis). Significant correlations are given with (*) = *P* < 0.05, (**) = *P* < 0.01.

### Immunoglobulin A

We used 1 g of wet feces and dried samples, balanced for the weight loss from drying similar as for the FGM extraction only that the weight of each samples corresponded to 1 g of fresh feces (for complete data, see Supplementary Table S2; for calculation for balancing weight loss from drying, see Supplementary File S1).

The particular masses for each dried sample were dissolved in 10 mL of PBS at pH 7.4. After shaking strongly, the samples were vortexed for 3 minutes and allowed to stand for 15 minutes. The shaking and vortexing procedure was repeated once. Afterwards, the samples were centrifuged for 20 minutes at 1600 g at room temperature. A supernatant of 800 μL was transferred into 1.5-mL tubes. Now, the 1.5-mL tube, containing the supernatant, was centrifuged for 15 minutes at 3260 g at room temperature. From this, a supernatant of 500 μL were transferred into 1.5-mL tubes and frozen at −20°C until analysis. IgA was determined using a Horse IgA ELISA Quantitation Kit (Cat. No. E70-116, Lot No. E70-116-14; Bethyl Laboratories, Inc.; https://www.bethyl.com/product/E70-116). The Elisa Kit was validated for the detection of IgA in horse serum and plasma. The company suggests that feces that contain horse IgA are suitable samples for the application of the Horse IgA ELISA Quantitation Kit. The order of the samples (CV of duplicates: <2%) on the two plates was the same as for FGMs.

### Statistical Analysis

FGM quantities are given in ng/g and IgA quantities in μg/g. The statistical analysis and the figures were done with the R-Project statistical environment, package R commander (R Development Core Team 2018). Some of the data (for complete data, see Supplementary Table S3) were not normally distributed (Shapiro–Wilk Test). Therefore, non-parametric generalized linear models (GLMs) were used for analysing the effect of the fixed factor ‘treatment’ on the FGM and IgA distributions (for complete GLM models, see Supplementary File S4). Thereafter, Spearman rank correlation tests were used to compare the preservation methods pairwise. Sequential Bonferroni corrections after Holm for multiple testing were applied to adjust the *P* values (for complete correlation data, see Supplementary File S4). All tests were two-tailed and the significance level was set at 0.05.

### Ethical considerations

The sampling person was not in contact with the animals from which she collected fecal samples. The non-invasive sampling of horses fecal samples did not cause the animals any harm, pain, or suffering (as defined in § 1, 3, and 7 of the German Animal Welfare Law) and did not require permission by the regional Animal Welfare Board, Tübingen.

## Results

### Speed of drying the fecal samples

The fecal samples (1.5 g) lost most of their humidity during the first 12 hours of drying, both when dried on air at room temperature (*n* = 6, weight loss median = 75%, min. = 73%, max. = 78%) and when dried on 20 mL of silica gel (*n* = 6, weight loss median = 75%, min. = 73%, max. = 76%). Only very little further reduction of the weight (i.e. humidity) was observed after drying the samples for 24 hours (*n* = 6; weight loss air drying: median = 77%, min. = 74%, max. = 79%; weight loss silica gel: median = 77.5%, min. = 77%, max. = 79%) and no further weight loss occurred afterwards (48 and 72 hours).

### FGM stability at room temperature and in dried samples

Individual animals varied in their fecal FGM concentrations (GLM: *n* = 72, SE = 0.43, *t* = 10.64, *P* < 0.001; Supplementary File S4). FGM concentrations did not differ significantly from concentrations in frozen samples when feces were air dried for 7 days or dried on silica gel for 1 or 7 days (GLM: *n* = 72, all *P* > 0.05, Fig. 2; File Supplementary S4). However, FGM quantities in samples dried on air for 1 day (GLM: *n* = 72, SE = 5.11, *t* = 1.74, *P* = 0.09) and those kept in wet samples at room temperature for 7 days (GLM: *n* = 72, SE = 5.11, *t* = −1.77, *P* = 0.08) tended to vary from those in frozen samples.

Furthermore, FGM concentrations in frozen samples correlated (Fig. 3) with those of the samples of both drying procedures after 1 and 7 days of drying (Spearman rank correlation test: *n* = 12, all *P* < 0.05; for full statistical data, see File Supplementary S4). In addition, FGM quantities correlated between both time points of air and silica gel drying (Spearman rank correlation test, *n* = 12, AD: *r*_s_ = 0.874, *P* = 0.003; SD: *r*_s_ = 0.853, *P* = 0.006). However, FGM concentrations in air-dried samples were not correlated with those of silica gel-dried ones (Spearman rank correlation test: *n* = 12, all *P* > 0.05).

### IgA stability at room temperature and in dried samples

**Figure 4:**
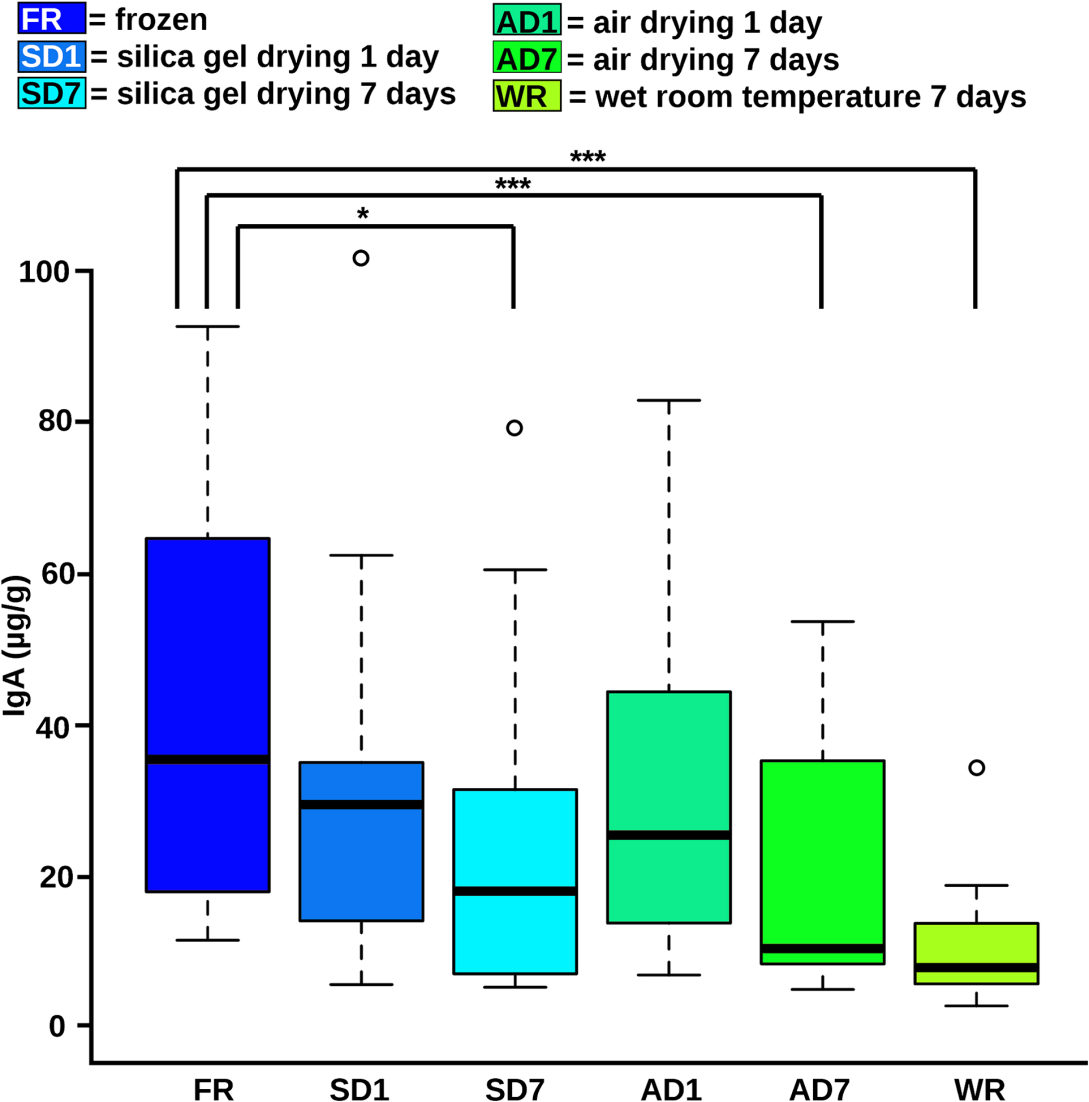
IgA concentrations. The boxes comprise 50% and each whisker 25% of the data of the particular preservation method. The line in the box represents the median and the circles above the box represent outliers. For full statistical data, see Supplementary File S4. *P < 0.5, ***P < 0.001.

Individuals also varied in their fecal IgA concentrations (GLM: *n* = 72, SE = 0.05, *t* = 9.65, *P* < 0.001). IgA concentrations (Fig. 4) did not differ significantly between frozen samples and samples dried for 1 day on air or on silica gel (GLM: *n* = 72, both *P* > 0.05). However, IgA concentrations were lower in samples dried 7 days on air (GLM: *n* = 72, SE = 0.59, *t* = −3.5, *P* < 0.001), in samples dried for 7 days in silica gel (GLM: *n* = 72, SE = 0.59, *t* = −2.63, *P* = 0.01), and in wet samples kept at room temperature for 7 days (GLM: *n* = 72, SE = 0.59, *t* = −5.14, *P* < 0.001) than in frozen samples.

**Figure 5:**
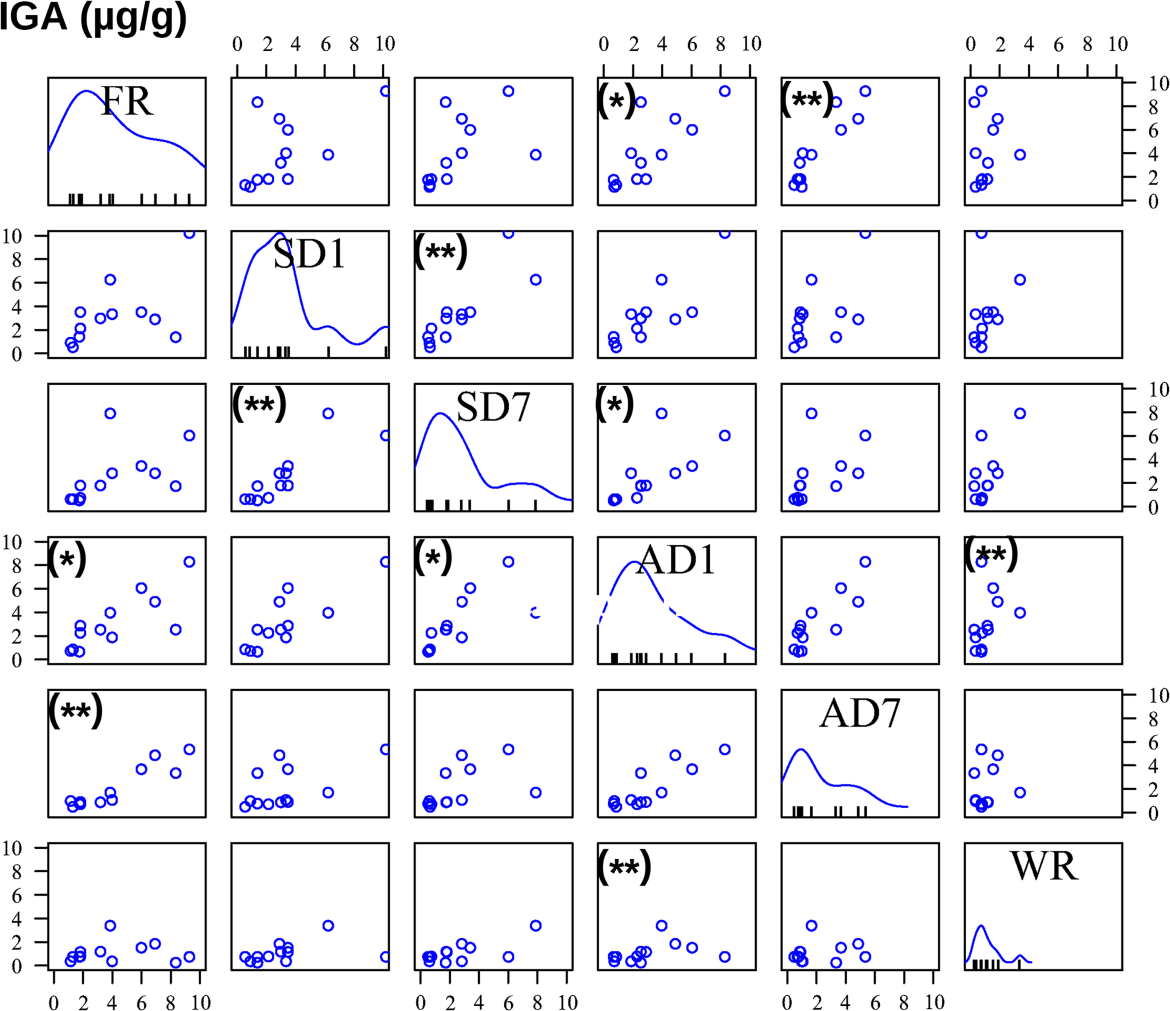
Correlations of IgA concentrations between preservation procedures. On the diagonal from the upper left to the lower right, the frequency distributions of IgA concentrations are shown for the 12 samples of each preservation procedure: FR = frozen samples, SD1 = silica gel-dried sample 1 day, SD7 = silica gel-dried samples 7 days, AD1 = air-dried sample 1 day, AD7 = air-dried samples 7 days, WR = wet sample at room temperature 7 days. IgA concentrations are given in μg/g for the *x* axis at each column. The 12 lines on the *x* axis within each preservation graph indicate the IgA concentration of each of the 12 samples. The scatterplots depict the correlations between IgA concentrations of pairs of preservation procedures (for full statistical data, see Supplementary File S4). For the correlations, *y* and *x* axes quantify the concentrations of IgA in the respective column (*x* axis) or row (*y* axis). Significant correlations are given with (^*^) = *P* < 0.05, (^**^) = *P* < 0.01.

Even though IgA quantities declined from frozen samples when they were dried on air for 1 and 7 days (Fig. 4), frozen sample quantities correlated well with those dried on air for 1 day (Spearman rank correlation test: *n* = 12, *r*_s_ = 0.769, *P* = 0.04) and for 7 days (Spearman rank correlation test: *n* = 12, *r*_s_ = 0.853, *P* = 0.005; Fig. 5; for full statistical data, see Supplementary File S4). IgA quantities in frozen samples did not correlate with those in silica gel-dried samples (Spearman rank correlation test: *n* = 12, *P* both > 0.05). However, IgA quantities tended to be correlated between both time points of air drying and correlated between both time points of silica gel drying (Spearman rank correlation test, *n* = 12, AD: *r*_s_ = 0.748, *P* = 0.06; SD: *r*_s_ = 0.860, *P* = 0.005). Finally, IgA concentrations in wet samples kept at room temperature for 7 days did not correlate with IgA concentrations in frozen samples or in samples from any of the drying procedures (Spearman rank correlation test: *n* = 12, all *P* > 0.05, Fig. 5).

## Discussion

### Fecal glucocorticoid metabolites

In the present study, bacterial decay of FGMs was prevented for up to 7 days when samples were air dried under controlled laboratory conditions or dried on silica gel under controlled conditions in an air tight tube. Only when wet fecal samples were kept for 7 days without drying, FGM concentrations declined. Thus, for FGM analysis, drying feces on air under controlled laboratory condition is convenient. However, when fresh samples cannot be frozen immediately or when drying samples on air is not applicable (e.g. in field studies), our results suggest that drying fecal samples on silica gel in air tight tubes is a reliable and convenient alternative to preserve FGMs. In a similar way, silica gel has also been used for the preservation of botanical specimens (Chase and Hills, 1991) and for samples for genetic analysis (Taberlet *et al.*, 1999; Murphy *et al.* 2002; Engelhardt *et al.*, 2017).

### Immunoglobulin A

When samples were air dried or dried on silica gel, an insignificant loss of IgA quantities occurred after 1 day, but a significant loss after 7 days. Keeping feces at room temperature without drying them is the least suitable method for measuring IgA, as this procedure produced the strongest decay in IgA concentrations and the sample quantities did not correlate well with the quantities in fresh samples or to the quantities in any of the drying procedures. We therefore suggest to use fresh samples (Zaine *et al.*, 2011) or to conserve fecal samples for IgA analysis through freezing (Hau *et al*., 2001) whenever possible. When field conditions do not allow for generating fresh or frozen samples, extrapolating the median loss of IgA through drying over a defined duration to the quantities expected for fresh samples has been suggested for air-dried human fecal samples (Vetvik *et al*., 1998). Drying horse fecal samples on air produced a reliable, quantifiable loss of IgA, as reduced quantities in the samples dried on air for one and for 7 days correlated well with quantities in the frozen samples. Drying the feces on silica gel reduced IgA decay, but only in some individuals as IgA quantities in silica gel drying could not be correlated with IgA quantities in frozen or air-dried samples.

### Influences on FGM and IgA preservation

FGM and IgA quantities differed between individuals as previously reported and because many factors influence their levels (FGM: Möstl *et al.*, 1999; Gorgasser *et al.*, 2007; Palme, 2019; IgA: May, 2007; Palm *et al.*, 2016). However, a diverse set of samples is advantageous for stability testing to be able to draw broad conclusions. The speed of drying was sufficient to prevent bacterial decay of FGMs (Möstl and Palme, 2002). Drying samples on air or on silica gel within 12 hours kept FGM concentrations in the samples stable.

The analysis of FGMs and IgA showed inconsistencies between preservation procedures, as some samples had even higher concentrations after running through preservation procedures than measured in the fresh samples. Since bacterial enzymes are mainly involved in the further metabolism of FGMs, the diversity of the individual gut microbiome may exert a strong influence here (Palme, 2019). Some metabolites may be better detected by the EIA than their precursors and this phenomenon can account for higher levels (e.g. Möstl *et al.*, 1999; Lexen *et al*., 2008). Furthermore, those inconsistencies could be explained by difficulties to establish equal fecal subsample compositions. Although the samples were well homogenized and visually homogenous, minor differences in amounts of undigested materials could not be excluded.

FGM and IgA degradation processes may differ between silica gel drying and air drying as FGM and IgA concentrations correlated well between time points within silica gel or air drying but did not correlate between the two preservation methods. While studies on mechanisms of FGM degradation during air drying were not conducted, possibly because of the satisfying stability of FGMs, Griebenow and Klibanov (1995) demonstrated an alteration of the secondary protein structure during dehydration of proteins in immunoglobulin G. Adding sorbitol and trehalose improved the storage stability of IgG after spray drying of protein solutions (Maury *et al.,* 2005). Future studies may evaluate whether adding sorbitol or trehalose enhances IgA preservation in fecal samples dried on silica gel. By applying ELISA kits validated specifically for the application to horse fecal samples, IgA detection may also be improved, while variability in IgA decay over time and preservation methods may remain.

## Conclusion

FGMs of horse feces dried on silica gel remained stable. Thus, drying samples on silica gel in air tight tubes adds to preserving fecal FGMs through freezing, air drying, or storage on alcohol (Palme *et al.*, 2013) and appears to be very helpful to preserve FGMs under field conditions, where freezing is not possible and changing humidity and temperature prevent air drying. However, for analysing immune responses to long lasting stress (Siegel, 1987; Herbert and Cohen, 1993), IgA preservation by freezing samples remains to be the most reliable method. IgA quantities in horse fecal samples dried on air can be extrapolated to the quantities in frozen samples, as reported for human fecal samples (Vetvik *et al.*, 1998).

## Supplementary Material

Supplementary_Data_coz065
