# Supplementary material for: Preservation of fecal glucocorticoid metabolites and immunoglobulin A through silica gel drying for field studies in horses
Source: Conserv Physiol. 2019 Oct 27;7(1):coz065. doi: 10.1093/conphys/coz065 (PMC6821355; doi:10.1093/conphys/coz065)
Supplement: Supplementary_Data_coz065 [file supplementary_data_coz065.pdf]

Supplementary Data

Preservation of fecal cortisol metabolites (FGM) and Immunoglobulin A (IgA) through silica gel drying for field studies in horses

Konstanze Krueger[1,2,] *, Isabell Marr[1,3], Andrea Dobler[1], Rupert Palme[4]

[1] Nuertingen-Geislingen University, Faculty Agriculture, Economics and Management, Department Equine Economics, Neckarsteige 6-10, 72622 Nürtingen, Germany [2] University of Regensburg, Zoology / Evolutionary Biology, Universitätsstraße 31, 93053 Regensburg, Germany
[3] University of Hohenheim, Behavioural Physiology of Farm Animals, Garbenstr. 17, 70599 Stuttgart, Germany
[4] University of Veterinary Medicine, Department of Biomedical Sciences, Veterinär-Platz 1, 1210 Vienna, Austria

* **Corresponding author:** Konstanze Krueger: Tel: +497022201331, Email: Konstanze.krueger@hfwu.de

Supplementary File S1

Calculation of sample weight – balancing weight loss through drying

FGMs

Glucocorticoid metabolites were extracted from horse feces with the simplified method described by Flauger *et al.*, (2010). We used 0.5 g of wet feces, which is equivalent to 33.33% of 1.5g wet feces from the frozen sample. In the dried samples we balanced the weight loss from drying, by weighting each sample within its filter and using the exact dry mass without the filter which, for FGM extraction, corresponded to 1.5 g of fresh feces in each sample (complete data see Table S1). For the calculation of the dry mass necessary for extraction, we applied the following formula:

weight dry sample for FGM extraction = weight dry feces * 33.33 / 100

Example calculation dry samples FGM (Supplementary Table S1):
original sample = weight original feces (1.51 g) + weight tee filter (0.25 g) = 1.76 g
weight dry sample = weight dry feces (0.26 g) + weight tee filter (0.25 g) = 0.51 g
weight dry feces = weight dry sample (0.51 g) - weight tee filter (0.25 g) = 0.26 g
weight loss = weight original feces (1.51 g) + weight tee filter (0.25 g) - weight dry feces (0.26) = 1.25g
% weight loss particular sample = weight loss 1.25 g * 100 / weight original feces (1.51 g) = 82,78%
weight dry sample for FGM extraction = weight dry feces (0.26) * 33.33 / 100 = 0.0867g

IgA

We used 1 g wet feces, which is equivalent to 66.67% of 1.5g wet feces from the frozen sample. In dried samples we balanced the weight loss from drying similar as for the FGM extraction only that the weight of each samples corresponded to 1 g of fresh feces (complete data see Table S1). We applied the following formula to calculate the dry mass that is necessary for the extraction:

weight dry sample for IgA extraction = weight dry feces * 66.67 / 100

Supplementary Table S2

FGM	horse number	horse 1	horse 2	horse 3	horse 4	horse 5	horse 6	horse 7	horse 8	horse 9	horse 10	horse 11	horse 12
1SD = 1 day silica gel drying	weight original feces	1,51	1,5	1,52	1,5	1,51	1,5	1,5	1,52	1,5	1,52	1,51	1,52
	weight tee filter	0,25	0,25	0,24	0,25	0,24	0,25	0,25	0,25	0,25	0,24	0,24	0,25
	weight dry feces + filter	0,51	0,64	0,54	0,61	0,53	0,61	0,56	0,5	0,57	0,52	0,58	0,5
	weight dry feces	0,26	0,39	0,3	0,36	0,29	0,36	0,31	0,25	0,32	0,28	0,34	0,25
	diffrence	1,25	1,11	1,22	1,14	1,22	1,14	1,19	1,27	1,18	1,24	1,17	1,27
	% loss	82,781	74	80,263	76	80,795	76	79,333	83,5526	78,667	81,579	77,4834	83,55263
	weight dry sample for extraction	0,0867	0,13	0,1	0,12	0,0967	0,12	0,1033	0,08333	0,1067	0,0933	0,11333	0,083333
7SD = 7 days silica gel drying	weight original feces	1,52	1,5	1,5	1,5	1,52	1,5	1,5	1,52	1,5	1,51	1,51	1,52
	weight tee filter	0,24	0,25	0,25	0,25	0,25	0,24	0,24	0,24	0,24	0,24	0,25	0,24
	weight dry feces + filter	0,51	0,62	0,52	0,6	0,52	0,6	0,54	0,49	0,54	0,48	0,58	0,48
	weight dry feces	0,27	0,37	0,27	0,35	0,27	0,36	0,3	0,25	0,3	0,24	0,33	0,24
	diffrence	1,25	1,13	1,23	1,15	1,25	1,14	1,2	1,27	1,2	1,27	1,18	1,28
	% loss	82,237	75,333	82	76,667	82,237	76	80	83,5526	80	84,106	78,1457	84,21053
	weight dry sample for extraction	0,09	0,1233	0,09	0,1167	0,09	0,12	0,1	0,08333	0,1	0,08	0,11	0,08
1AD = 1 day air drying	weight original feces	1,5	1,5	1,52	1,51	1,51	1,5	1,5	1,51	1,5	1,51	1,52	1,51
	weight airtight tube	84,03	74,96	74,35	83,94	76,54	82,52	79,32	84,81	38,17	81,36	9,34	37,82
	weight dry feces + airtight tube	84,35	75,36	74,68	84,29	76,86	82,9	79,64	85,1	38,49	81,67	9,68	38,13
	weight dry feces	0,32	0,4	0,33	0,35	0,32	0,38	0,32	0,29	0,32	0,31	0,34	0,31
	diffrence	1,18	1,1	1,19	1,16	1,19	1,12	1,18	1,22	1,18	1,2	1,18	1,2
	% loss	78,667	73,333	78,289	76,821	78,808	74,667	78,667	80,7947	78,667	79,47	77,6316	79,4702
	weight dry sample for extraction	0,1067	0,1333	0,11	0,1167	0,1067	0,1267	0,1067	0,09667	0,1067	0,1033	0,11333	0,103333
7AD = 7 days air drying	weight original feces	1,51	1,5	1,52	1,51	1,51	1,5	1,5	1,52	1,5	1,5	1,51	1,5
	weight airtight tube	74,96	79,56	82	73,96	81,14	84,94	77,83	82,15	37,01	76,7	8,3	76,02
	weight dry feces + airtight tube	75,31	79,97	82,34	74,34	81,46	85,32	78,15	82,5	37,31	77	8,62	76,31
	weight dry feces	0,35	0,41	0,34	0,38	0,32	0,38	0,32	0,35	0,3	0,3	0,32	0,29
	diffrence	1,16	1,09	1,18	1,13	1,19	1,12	1,18	1,17	1,2	1,2	1,19	1,21
	% loss	76,821	72,667	77,632	74,834	78,808	74,667	78,667	76,9737	80	80	78,8079	80,66667
	weight dry sample for extraction	0,1167	0,1367	0,1133	0,1267	0,1067	0,1267	0,1067	0,11667	0,1	0,1	0,10667	0,096667
WR = wet sample room temperature	weight original feces	1,51	1,5	1,52	1,53	1,52	1,5	1,5	1,52	1,5	1,5	1,51	1,51
	weight petri dish	14,84	14,78	15,26	14,9	14,87	14,89	14,81	14,75	14,93	14,75	14,81	15,21
	weight dry feces + petri dish	no difference to original feces weight + weight tee filter											
	weight dry feces												
	diffrence												
	% loss												
	weight sample for extraction	equal to original feces weight											

IgA	horse number	horse 1	horse 2	horse 3	horse 4	horse 5	horse 6	horse 7	horse 8	horse 9	horse 10	horse 11	horse 12
1SD = 1 day silica gel drying	weight original feces	1,52	1,5	1,5	1,52	1,51	1,5	1,5	1,51	1,5	1,52	1,5	1,5
	weight tee filter	0,25	0,25	0,24	0,24	0,24	0,25	0,24	0,25	0,24	0,24	0,24	0,25
	weight dry feces + filter	0,56	0,63	0,52	0,61	0,53	0,62	0,57	0,54	0,61	0,51	0,55	0,47
	weight dry feces	0,31	0,38	0,28	0,37	0,29	0,37	0,33	0,29	0,37	0,27	0,31	0,22
	diffrence	1,21	1,12	1,22	1,15	1,22	1,13	1,17	1,22	1,13	1,25	1,19	1,28
	% loss	79,605	74,667	81,333	75,658	80,795	75,333	78	80,7947	75,333	82,237	79,3333	85,33333
	weight dry sample for extraction	0,2067	0,2533	0,1867	0,2467	0,1933	0,2467	0,22	0,19333	0,2467	0,18	0,20667	0,146667
7SD = 7 days silica gel drying	weight original feces	1,52	1,5	1,51	1,5	1,51	1,5	1,5	1,5	1,5	1,51	1,5	1,52
	weight tee filter	0,24	0,25	0,25	0,25	0,25	0,24	0,24	0,24	0,24	0,25	0,25	0,24
	weight dry feces + filter	0,53	0,62	0,53	0,62	0,51	0,6	0,55	0,54	0,56	0,51	0,52	0,47
	weight dry feces	0,29	0,37	0,28	0,37	0,26	0,36	0,31	0,3	0,32	0,26	0,27	0,23
	diffrence	1,23	1,13	1,23	1,13	1,25	1,14	1,19	1,2	1,18	1,25	1,23	1,29
	% loss	80,921	75,333	81,457	75,333	82,781	76	79,333	80	78,667	82,781	82	84,86842
	weight dry sample for extraction	0,1933	0,2467	0,1867	0,2467	0,1733	0,24	0,2067	0,2	0,2133	0,1733	0,18	0,153333
1AD = 1 day air drying	weight original feces	1,51	1,5	1,52	1,5	1,51	1,5	1,5	1,52	1,5	1,52	1,52	1,51
	weight airtight tube	82,97	75,3	82,74	75,77	83,96	70,43	78,28	85,75	78,06	77,45	9,36	8,3
	weight dry feces + airtight tube	83,3	75,68	83,09	76,12	84,27	70,8	78,6	86,06	78,37	77,71	9,69	8,59
	weight dry feces	0,33	0,38	0,35	0,35	0,31	0,37	0,32	0,31	0,31	0,26	0,33	0,29
	diffrence	1,18	1,12	1,17	1,15	1,2	1,13	1,18	1,21	1,19	1,26	1,19	1,22
	% loss	78,146	74,667	76,974	76,667	79,47	75,333	78,667	79,6053	79,333	82,895	78,2895	80,7947
	weight dry sample for extraction	0,22	0,2533	0,2333	0,2333	0,2067	0,2467	0,2133	0,20667	0,2067	0,1733	0,22	0,193333
7AD = 7 days air drying	weight original feces	1,52	1,5	1,52	1,52	1,51	1,5	1,5	1,52	1,5	1,51	1,53	1,5
	weight airtight tube	80,11	73,7	79,83	87,22	82,55	77,51	76,07	97,4	47,81	79,97	8,3	47,16
	weight dry feces + airtight tube	80,44	74,1	80,17	87,61	82,92	77,86	76,41	97,71	48,15	80,26	8,64	47,47
	weight dry feces	0,33	0,4	0,34	0,39	0,37	0,35	0,34	0,31	0,34	0,29	0,34	0,31
	diffrence	1,19	1,1	1,18	1,13	1,14	1,15	1,16	1,21	1,16	1,22	1,19	1,19
	% loss	78,289	73,333	77,632	74,342	75,497	76,667	77,333	79,6053	77,333	80,795	77,7778	79,33333
	weight dry sample for extraction	0,22	0,2667	0,2267	0,26	0,2467	0,2333	0,2267	0,20667	0,2267	0,1933	0,22667	0,206667
WR = wet sample room temperature	weight original feces	1,5	1,5	1,51	1,5	1,5	1,5	1,5	1,51	1,5	1,52	1,52	1,52
	weight petri dish	14,81	14,82	14,76	14,85	14,82	14,9	14,83	14,82	14,86	14,82	14,85	14,91
	weight dry feces + petri dish	no difference to original feces weight + weight tee filter											
	weight dry feces												
	diffrence												
	% loss												
	weight sample for extraction	equal to original feces weight											

Supplementary Table S3

horse_num	treatment	FGM ng/g	IgA µg/g	horse_num	treatment	FGM ng/g	IgA µg/g
1	FR	26,62	3,87	7	FR	37,80	1,81
1	SD1	26,20	6,23	7	SD1	51,50	3,49
1	SD7	15,88	7,89	7	SD7	53,28	1,79
1	AD1	19,80	3,95	7	AD1	46,88	2,87
1	AD7	19,32	1,68	7	AD7	42,50	0,90
1	WR	16,15	3,39	7	WR	34,12	1,16
2	FR	33,57	8,33	8	FR	22,29	6,93
2	SD1	30,36	1,39	8	SD1	16,30	2,88
2	SD7	30,56	1,71	8	SD7	15,05	2,84
2	AD1	42,86	2,52	8	AD1	33,86	4,90
2	AD7	35,51	3,34	8	AD7	39,05	4,86
2	WR	0,00	0,26	8	WR	16,32	1,86
3	FR	16,17	1,74	9	FR	32,23	3,18
3	SD1	14,85	1,39	9	SD1	58,23	2,98
3	SD7	16,54	0,51	9	SD7	43,30	1,77
3	AD1	18,96	0,67	9	AD1	33,24	2,53
3	AD7	16,04	0,78	9	AD7	34,19	0,86
3	WR	15,80	0,75	9	WR	63,45	1,18
4	FR	41,93	4,00	10	FR	46,95	1,30
4	SD1	34,76	3,33	10	SD1	35,57	0,54
4	SD7	42,68	2,84	10	SD7	27,13	0,64
4	AD1	52,58	1,86	10	AD1	76,93	0,86
4	AD7	53,44	1,06	10	AD7	37,84	0,48
4	WR	17,09	0,36	10	WR	20,57	0,76
5	FR	26,55	9,27	11	FR	75,15	5,98
5	SD1	29,96	10,20	11	SD1	96,32	3,49
5	SD7	26,51	6,04	11	SD7	74,69	3,42
5	AD1	26,95	8,29	11	AD1	63,01	6,06
5	AD7	27,39	5,35	11	AD7	57,90	3,68
5	WR	12,52	0,75	11	WR	89,19	1,53
6	FR	57,49	1,80	12	FR	30,73	1,13
6	SD1	57,31	2,14	12	SD1	22,35	0,91
6	SD7	55,62	0,73	12	SD7	33,35	0,60
6	AD1	82,87	2,25	12	AD1	55,86	0,72
6	AD7	85,95	0,71	12	AD7	46,97	0,99
6	WR	27,26	0,78	12	WR	26,33	0,34

Supplementary File S

Full Statistical Data

GLM and Correlation for FGM Analysis

Shapiro-Wilk normality test

data: FGM

W = 0.93117, p-value = 0.0007006

Call:

glm(formula = FGM ~ horse + treatment, family = gaussian(identity),
    data = Dataset)

Deviance Residuals:

Min	1Q	Median	3Q	Max
-24.271	-7.410	0.246	4.475	35.957

Coefficients:

	Estimate	Std. Error	t value	Pr(>|t|)	
(Intercept)	7.747	4.554	1.701	0.0937	.
Horse	4.545	0.427	10.644	7.02e-16	***
treatment[T.AD1]	8.861	5.106	1.735	0.0874	.
treatment[T.AD7]	4.052	5.106	0.793	0.4304	
treatment[T.WR]	-9.057	5.106	-1.774	0.0808	.
treatment[T.SD1]	2.187	5.106	0.428	0.6698	
treatment[T.SD7]	-1.074	5.106	-0.210	0.8340	

Signif. codes: 0 '***' 0.001 '**' 0.01 '*' 0.05 '.' 0.1 ' ' 1

(Dispersion parameter for gaussian family taken to be 156.4348)

Null deviance: 30037 on 71 degrees of freedom

Residual deviance: 10168 on 65 degrees of freedom

AIC: 576.75

Spearman rank correlations FGM:

	FR	AD1	AD7	WR	SD1	SD7
FR	1.0000	0.8531	0.7483	0.6154	0.8462	0.8112
AD1	0.8531	1.0000	0.8741	0.5594	0.5664	0.6503
AD7	0.7483	0.8741	1.0000	0.6154	0.5175	0.7133
WR	0.6154	0.5594	0.6154	1.0000	0.7343	0.7692

SD1	0.8462	0.5664	0.5175	0.7343	1.0000	0.8531
SD7	0.8112	0.6503	0.7133	0.7692	0.8531	1.0000

Number of observations: 12

Pairwise two-sided p-values:

	FR	AD1	AD7	WR	SD1	SD7
FR		0.0004	0.0051	0.0332	0.0005	0.0014
AD1	0.0004		0.0002	0.0586	0.0548	0.0220
AD7	0.0051	0.0002		0.0332	0.0849	0.0092
WR	0.0332	0.0586	0.0332		0.0065	0.0034
SD1	0.0005	0.0548	0.0849	0.0065		0.0004
SD7	0.0014	0.0220	0.0092	0.0034	0.0004	

Adjusted p-values (Holm's method)

	FR	AD1	AD7	WR	SD1	SD7
FR		0.0059	0.0461	0.1658	0.0063	0.0150
AD1	0.0059		0.0030	0.1658	0.1658	0.1322
AD7	0.0461	0.0030		0.1658	0.1658	0.0644
WR	0.1658	0.1658	0.1658		0.0523	0.0345
SD1	0.0063	0.1658	0.1658	0.0523		0.0059
SD7	0.0150	0.1322	0.0644	0.0345	0.0059	

GLM and Correlation for IgA Analysis

Shapiro-Wilk normality test

data: IgA

W = 0.84018, p-value = 0.0000002461

Call:

glm(formula = IgA ~ horse_data_ranking + treatment, family = gaussian(identity),
    data = Dataset)

Deviance Residuals:

Min	1Q	Median	3Q	Max
-2.9575	-0.8581	-0.1246	0.7106	4.3451

Coefficients:

	Estimate	Std. Error	t value	Pr(>|t|)	
(Intercept)	1.02740	0.52463	1.958	0.054486	.
horse_data_ranking	0.47478	0.04919	9.652	3.58e-14	***
treatment[T.AD1]	-0.99085	0.58823	-1.684	0.096888	.
treatment[T.AD7]	-2.05656	0.58823	-3.496	0.000856	***
treatment[T.WR]	-3.02078	0.58823	-5.135	2.77e-06	***
treatment[T.SD1]	-0.86603	0.58823	-1.472	0.145775	
treatment[T.SD7]	-1.54685	0.58823	-2.630	0.010657	*

Signif. codes: 0 '***' 0.001 '**' 0.01 '*' 0.05 '.' 0.1 ' ' 1

(Dispersion parameter for gaussian family taken to be 2.07608)

    Null deviance: 394.24 on 71 degrees of freedom
Residual deviance: 134.95 on 65 degrees of freedom
AIC: 265.56

Number of Fisher Scoring iterations: 2

Spearman rank correlations IgA:

	FR	WR	AD1	SD1	AD7	SD7
FR	1.0000	0.0769	0.7692	0.5594	0.8531	0.7413
WR	0.0769	1.0000	0.5315	0.4476	0.0769	0.5175
AD1	0.7692	0.5315	1.0000	0.7692	0.7483	0.8881
SD1	0.5594	0.4476	0.7692	1.0000	0.5385	0.8601
AD7	0.8531	0.0769	0.7483	0.5385	1.0000	0.7413
SD7	0.7413	0.5175	0.8881	0.8601	0.7413	1.0000

Number of observations: 12

Pairwise two-sided p-values:

	FR	WR	AD1	SD1	AD7	SD7
FR		0.8122	0.0034	0.0586	0.0004	0.0058
WR	0.8122		0.0754	0.1446	0.8122	0.0849
AD1	0.0034	0.0754		0.0034	0.0051	0.0001
SD1	0.0586	0.1446	0.0034		0.0709	0.0003
AD7	0.0004	0.8122	0.0051	0.0709		0.0058
SD7	0.0058	0.0849	0.0001	0.0003	0.0058	

Adjusted p-values (Holm's method)

	FR	WR	AD1	SD1	AD7	SD7
FR		1.0000	0.0414	0.4101	0.0054	0.0522
WR	1.0000		0.4254	0.4338	1.0000	0.4254
AD1	0.0414	0.4254		0.0414	0.0512	0.0017
SD1	0.4101	0.4338	0.0414		0.4254	0.0046
AD7	0.0054	1.0000	0.0512	0.4254		0.0522
SD7	0.0522	0.4254	0.0017	0.0046	0.0522	
